# Correlation of Plasma Amino Acid and Anthropometric Profiles with Brown Adipose Tissue Density in Humans

**DOI:** 10.3390/jcm10112339

**Published:** 2021-05-27

**Authors:** Miyuki Kuroiwa, Sayuri Hamaoka-Fuse, Masahiro Sugimoto, Yuko Kurosawa, Yasuko Aita, Atsumi Tomita, Mikiko Anjo, Riki Tanaka, Tasuki Endo, Ryotaro Kime, Takafumi Hamaoka

**Affiliations:** 1 Department of Sports Medicine for Health Promotion, Tokyo Medical University, Tokyo 160-8402, Japan; kuroiwa@tokyo-med.ac.jp (M.K.); fuse@tokyo-med.ac.jp (S.H.-F.); kurosawa@tokyo-med.ac.jp (Y.K.); anjo@tokyo-med.ac.jp (M.A.); s118042@tokyo-med.ac.jp (R.T.); endo@tokyo-med.ac.jp (T.E.); kime@tokyo-med.ac.jp (R.K.); 2 Research and Development Center for Minimally Invasive Therapies, Institute of Medical Science, Tokyo Medical University, Tokyo 160-8402, Japan; mshrsgmt@tokyo-med.ac.jp (M.S.); ait_18y@tokyo-med.ac.jp (Y.A.); tomita@tokyo-med.ac.jp (A.T.)

**Keywords:** amino acid concentrations, brown adipose tissue, plasma, biomarker, anthropometric parameters

## Abstract

This study examined the relationship between plasma amino acid (AA) concentrations, including branched-chain AAs, and brown adipose tissue density (BAT-d). One hundred and seventy-three subjects (69 men, 104 women) aged 22–68 years were recruited during the winter season. AAs were comprehensively quantified using liquid chromatography-time-of-flight-mass spectrometry. The total hemoglobin concentration in the supraclavicular region ([total-Hb]sup), an indicator of BAT-d, was assessed using near-infrared time-resolved spectroscopy. Anthropometric parameters, including age, percentage of body fat, and visceral fat, were evaluated. Factors associated with higher (≥74 µM) or lower (<74 µM) [total-Hb]sup were investigated by multiple logistic regression models that included AA concentrations alone (model 1) or AA concentrations and anthropometric parameters (model 2) as independent variables. When adjusted for the false discovery rate, [total-Hb]sup was positively correlated with glycine and asparagine levels in men and with the serine level in both men and women and was negatively correlated with the branched-chain AA concentration in men. Models 1 and 2 correlated with higher or lower BAT-d for men (*r* = 0.73, *p* = 0.015) and women (*r* = 0.58, *p* = 0.079) and for men (*r* = 0.82, *p* = 0.0070) and women (*r* = 0.70, *p* = 0.020), respectively. A combination of anthropometric parameters and plasma AA concentrations could be a reliable biomarker for higher and lower BAT-d.

## 1. Introduction

Brown adipose tissue (BAT) dissipates heat and secretes anti-inflammatory cytokines, known as BATkines, and it is a potential strategy for promoting cardiometabolic health [1,2]. BAT is reported to be related to less adiposity, including the percentage of whole body fat (%BF) and visceral fat area (VFA) [3,4,5], in healthy individuals and to increased glucose sensitivity in their obese counterparts [6] and patients with type 2 diabetes [7]. Therefore, increasing the activity or volume of BAT may help to combat obesity and certain chronic diseases, such as type 2 diabetes mellitus. The activity of BAT can be evaluated by ^18^F-fluorodeoxyglucose (FDG)-positron emission tomography (PET)/computed tomography (CT) (^18^FDG-PET/CT) in cold-stimulated environments [3,8,9]. Other non-invasive technologies used to evaluate the characteristics of BAT in humans include magnetic resonance imaging [10], skin thermal measurements [11], infrared thermography [12], near-infrared time-resolved spectroscopy (NIR_TRS_) [13,14], and contrast-enhanced ultrasonography [15]. However, each method has limitations, including high cost, ionizing radiation exposure, being time-intensive to perform, and/or requirement for acute cold exposure to stimulate BAT [3,8,9].

Clinically relevant biomarkers that can be used to evaluate the activity of BAT in individual subjects have long been sought. BAT mainly utilizes fatty acids as a primary energy source and glucose as a secondary source for non-shivering thermogenesis [8,16]. A recent study demonstrated that BAT contributes to the utilization of certain serum amino acids (AAs), such as valine and leucine, which are known as branched-chain AAs (BCAAs), especially in subjects with high BAT following 2 h of cold exposure [17]. However, that study included a small sample size comprised only of men and did not investigate thermoneutral BCAA profiles related to the activity of BAT in blood.

In this study, NIR_TRS_ was used to monitor the vascular density of BAT (BAT-d), given that microvascular beds, evaluated based on the total hemoglobin (Hb) concentration in the supraclavicular region ([total-Hb]sup), are more abundant in BAT than in white adipose tissue [13].The reliability of [total-Hb]sup compared with BAT activity/volume parameters determined using cold-induced FDG-PET/CT measurements has been confirmed in previous studies [13,14,18].

In this research, we were attempting to find a clinically practical and simple biomarker for BAT-d without acute cold exposure, which is needed for ^18^FDG-PET/CT measurements. We hypothesized that the plasma AA profile would correlate with [total-Hb]sup and would be a practical biomarker in the clinical setting. Therefore, this study examined the relationship between the plasma AA profile under thermoneutral conditions (normal room temperature) and NIR_TRS_-determined BAT-d in a large sample of men and women.

## 2. Materials and Methods

### 2.1. Participants and Diagnosis

The study participants were recruited by placing advertisements on posters, the Internet, or via direct contact. A total of 241 volunteers were recruited, 68 of whom were excluded because they were pregnant (*n* = 15) or refused to provide a blood sample (*n* = 53), leaving 173 subjects (69 men, 104 women, aged 22–68 years) for enrollment in the study. When each subject arrived in the laboratory, where the room temperature was regulated at 23–27 °C, the following parameters were measured: body height, body weight, %BF, VFA, subcutaneous adipose tissue thickness (SATsup) in the supraclavicular region, BAT-d, and plasma AA concentrations. 

The study design and protocols were approved by the institutional review board of Tokyo Medical University (approval no. 2017–199 and 2019–0028) and conducted in accordance with the ethical principles of the Declaration of Helsinki. Written informed consent was obtained from all study participants. The study was conducted during the winter season (December to April) between 2018 and 2020.

### 2.2. Measurement of Anthropometric Parameters

Body mass index (BMI) was calculated as body weight in kilograms divided by the square of height in meters (kg/m^2^). The %BF was estimated using the multifrequency bioelectric impedance method (Inbody 720 Body Composition Analyzer; InBody Japan, Tokyo, Japan). VFA was estimated using bioelectrical impedance analysis (EW-FA90; Panasonic, Osaka, Japan). The SATsup was measured by B-mode ultrasonography (Vscan Dual Probe; GE Vingmed Ultrasound AS, Horten, Norway) using the attached distance measuring system and calculated as the mean value of two measurements. 

BAT-d, evaluated by the [total-Hb]sup using NIR_TRS_ (TRS-20; Hamamatsu Photonics K.K., Hamamatsu, Japan), was measured for 1 min at room temperature (23–27 °C). The probes were placed on the skin in the supraclavicular region, which potentially contains BAT. Participants were required to remain seated in an upright position during the measurements, as previously described [13,19]. NIR wavelengths of 760, 800, and 830 nm were used to evaluate the concentrations of oxygenated Hb, deoxygenated Hb, and total Hb. With the 3-cm probe used in this study, light can reach a mean depth of 2 cm [20], where a certain amount of BAT may be located [21].

The tissue was illuminated using a 200-µm core diameter optical fiber by pulsed light generated from ps light pulses, with 100 ps full width at half-maximum, a 5-MHz repetition rate, and an average power of 80 µW for each wavelength. The emitted photons migrated into the tissue and were reflected by an optical bundle fiber (3-mm diameter), through which they were transferred to a photomultiplier tube for single-photon detection and processing. After the digitized temporal profile, data were fitted according to photon diffusion theory, the absorption coefficient and reduced scattering coefficient values at 760, 800, and 830 nm were obtained using the least-squares fitting method. The absolute total Hb concentration was calculated as the sum of the oxygenated and deoxygenated Hb concentrations [22]. The NIR_TRS_ system collected data every 10 s. The coefficient of variation for repeated measurements of total Hb concentration was 4.9% [13].

In this study, the [total-Hb]sup was adjusted according to the underlying adipose tissue thickness [23]. Our previous study indicated that a cut-off [total-Hb]sup value of 74.0 µM was best for distinguishing higher BAT-d from lower BAT-d, as evaluated by ^18^FDG-PET/CT, with an accuracy of 82.8%, a sensitivity of 75.0%, a specificity of 100%, a positive predictive value of 100%, and a negative predictive value of 64.3% [13].

### 2.3. Sample Collection

Venous blood was collected from the 173 subjects and incubated for 30 min at room temperature (24 °C). Then, the plasma was collected by centrifugation for 15 min at 1000× *g* and stored at −80 °C until needed for metabolomic analyses.

### 2.4. Amino Acid Profiling

Sample processing and AA analyses were performed as described in our previous study [24] with some modifications. We measured the processed samples using only the positive mode to profile the AAs. Each plasma sample (10 μL) was mixed with methanol (90 μL) containing 1.5 μM of a standard compound. Following centrifugation at 20,380× *g* for 10 min at 4 °C, 90 μL of the supernatant were transferred to a fresh tube and vacuum-dried. The sample was reconstituted with 90% methanol (10 μL) and water (190 μL), vortexed, and centrifuged at 20,380× *g* for 10 min at 4 °C. For liquid chromatography-time-of-flight mass spectrometry (LC-TOF-MS) analysis, 1 μL of each supernatant was injected into the system.

The LC-TOF-MS instrument [19] and its parameters [24] have been described elsewhere. Briefly, a 1290 Infinity LC system and G6230B time-of-flight MS (TOF-MS; Agilent Technologies, Santa Clara, CA, USA) were used. Chromatographic separation was performed using an ACQUITY BEH C18 column (2.1 mm i.d. × 50 mm, 1.7 μm; Waters, Milford, MA, USA) at 40 °C.

Peaks derived from the AAs and internal standards were analyzed using MassHunter Qualitative Analysis software (version B.08.00; Agilent Technologies). The peaks were automatically integrated by the software but were also curated manually by the analysts. The peak areas of the AAs were divided by one of the internal standards (d_6_-*N*_1_,*N*_8_-diacetylspermidine) to yield relative areas. The absolute concentrations of each AA were calculated by comparing the relative area of each sample with that of the standard mixture.

### 2.5. Data Analysis

The relationship between BAT-d and the quantified AA values was evaluated. The BCAAs were evaluated together and for leucine, isoleucine, and valine individually. To eliminate fluctuation in the AA concentrations in each subject, the absolute concentration was divided by the total AA concentration, yielding the relative concentration. Relative concentrations were used for all analyses. These anthropometric parameters were categorized lower and higher groups using cut-off values for obesity [25]; BMI was divided by 25, %BF was divided by 25 in men and 30 in women, and VFA was divided by 100.

Spearman correlation coefficients were calculated for BAT-d and for the concentration of each AA or BCAA. In view of the multiple independent tests, *p*-values were adjusted using the false discovery rate method (Benjamini and Hochberg) to yield *Q*-values. Multiple logistic regression analyses (MLRs) were developed using the forced entry method to discriminate higher (≥74 µM) and lower (<74 µM) [total-Hb]sup using [total-Hb]sup as the dependent variable and the concentration of each plasma AA as the independent variable and using [total-Hb]sup as the dependent variable and age, %BF, VFA, and each plasma AA as independent variables. Receiver-operating characteristic (ROC) curves were analyzed by plotting true-positive rates (sensitivity) against false-positive rates (1 − specificity) to compare the discriminatory accuracy of [total-Hb]sup for survival and event-free survival.

All statistical analyses were performed using IBM SPSS Statistics version 27 (IBM Japan, Tokyo, Japan).

## 3. Results

The subject characteristics are summarized in Table 1. The height, weight, body mass index, and VFA were significantly higher, and %BF was significantly lower in men than in women. No significant sex-related differences were found in age or [total-Hb]sup.

**Figure 1:**
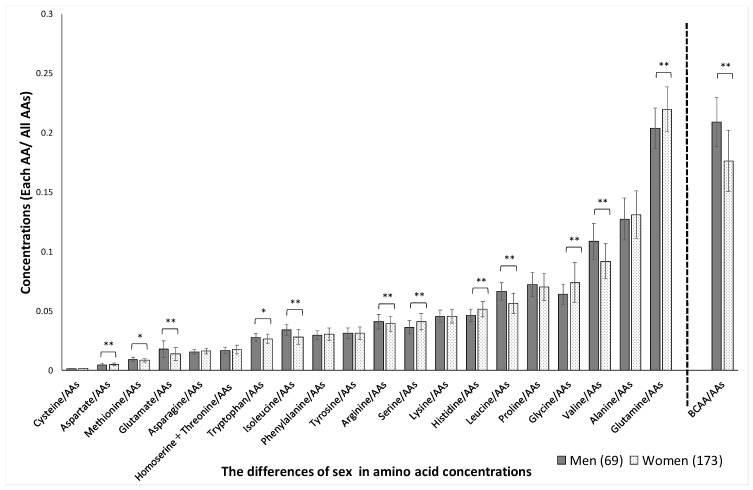
Sex-related differences in amino acid concentrations. The absolute concentration was divided by the total AA concentration, yielding the relative concentration. AAs, amino acids; BCAAs, branched-chain amino acids. * *p* < 0.05, ** *p* < 0.01 in men versus women.

**Figure 2:**
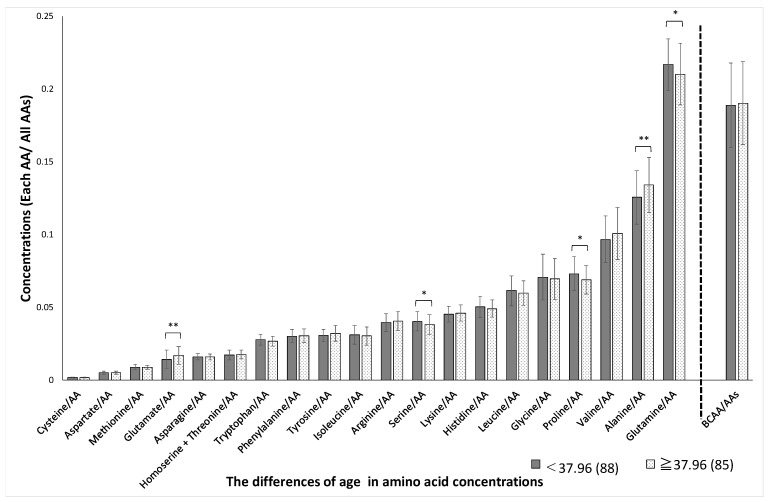
Age-related differences in amino acid concentrations. The absolute concentration was divided by the total AA concentration, yielding the relative concentration. Participants were divided into two groups based on age, with a median of 37.96 years. AAs, amino acids; BCAAs, branched-chain amino acids. * *p* < 0.05, ** *p* < 0.01 in age < 37.96 versus age ≥ 37.96.

**Figure 3:**
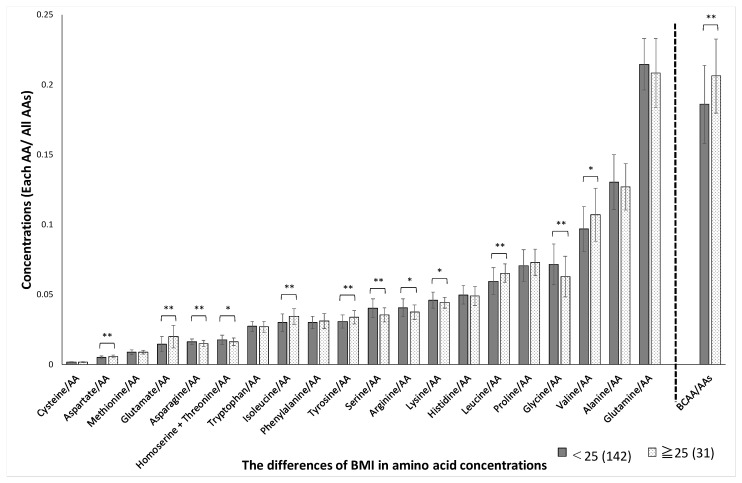
BMI-related differences in amino acid concentrations. The absolute concentration was divided by the total AA concentration, yielding the relative concentration. Participants were divided into two groups based on BMI 25. AAs, amino acids; BCAAs, branched-chain amino acids BCAAs, branched-chain amino acids. * *p* < 0.05, ** *p* < 0.01 in BMI < 25 versus BMI ≥ 25.

**Figure 4:**
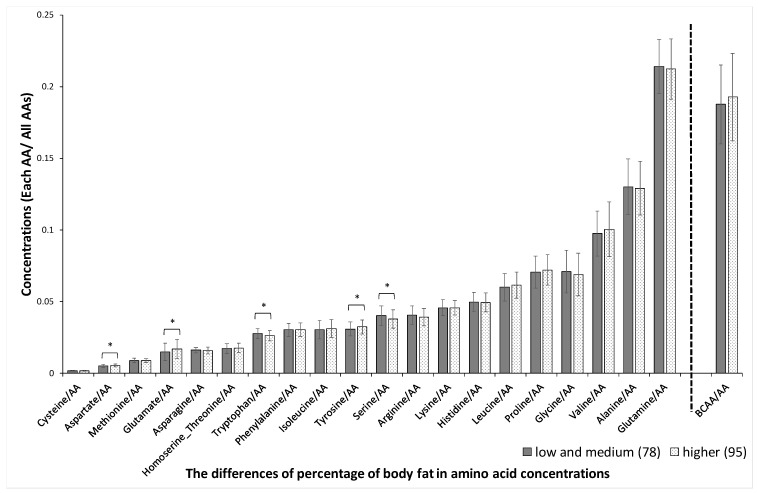
Body fat percentage-related differences in amino acid concentrations. The absolute concentration was divided by the total AA concentration, yielding the relative concentration. Participants were divided into two groups based on body fat percentage, 25% in men and 30% in women. AAs, amino acids; BCAAs, branched-chain amino acids. * *p* < 0.05, ** *p* < 0.01 in body fat percentage < 25% versus body fat percentage ≥ 25% in men and in body fat percentage < 30% versus body fat percentage ≥ 30% in women.

**Figure 5:**
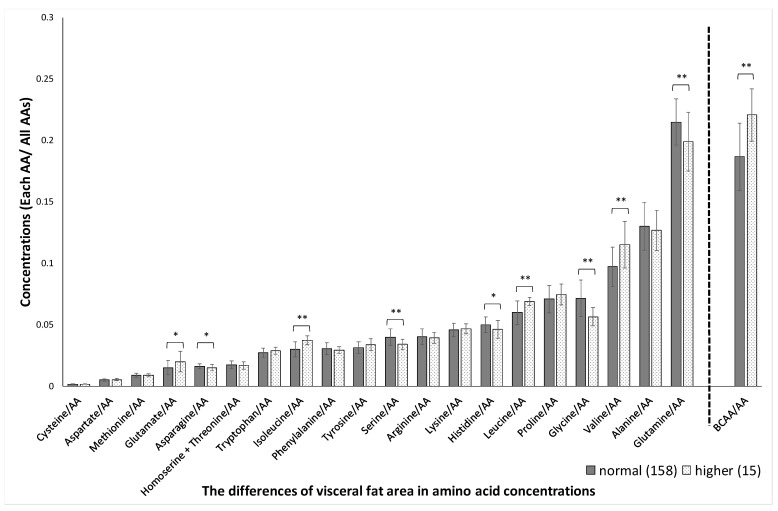
Visceral fat area-related differences in amino acid concentrations. The absolute concentration was divided by the total AA concentration, yielding the relative concentration. Participants were divided into two groups based on the visceral fat area 100. AAs, amino acids; BCAAs, branched-chain amino acids. * *p* < 0.05, ** *p* < 0.01 in visceral fat area < 100 cm^2^ versus ≥ 100 cm^2^.

Significant sex-related differences were found in the concentrations of aspartate, methionine, glutamate, tryptophan, isoleucine, arginine, serine, histidine, leucine, glycine, valine, glutamine, and BCAAs, as shown in Figure 1. The variation (SD) in plasma amino acid concentrations between men and women was not significantly different (*p* = 0.53). Significant age-related differences were found in the concentrations of glutamate, serine, proline, alanine, and glutamine, as shown in Figure 2. Significant BMI-related differences were found in the concentrations of aspartate, glutamate, asparagine, homoserine + threonine, isoleucine, tyrosine, serine, arginine, lysine, leucine, glycine, valine, and BCAA, as shown in Figure 3. Significant %BF-related differences were found in the concentrations of aspartate, glutamate, tryptophan, tyrosine, and serine, as shown in Figure 4. Significant VFA related differences were found in the concentration of glutamate, asparagine, isoleucine, serine, histidine, leucine. glycine, valine, glutamine, and BCAA, as shown in Figure 5.

The correlations between the AA concentrations and [total-Hb]sup are shown in Table 2. In men, there were significant positive correlations of glycine (*r* = 0.39, *p =* 0.00095, *Q* = 0.005), serine (*r* = 0.38, *p =* 0.0012, *Q* = 0.0050), and asparagine (*r* = 0.35, *p =* 0.0035, *Q* = 0.012) with [total-Hb]sup and a negative correlation of the BCAA concentration with [total-Hb]sup (*r* = −0.30, *p =* 0.014, *Q* = 0.045). In women, the serine concentration (*r* = 0.31, *p =* 0.016, *Q* = 0.019) correlated positively with [total-Hb]sup.

The correlation between [total-Hb]sup and age was not significant (*r* = −0.11, *p* = 0.161). The correlations between the AA concentrations and age were significant for alanine/AA (*r* = 0.21, *p =* 0.0061), serine/AA (*r* = −0.19, *p =* 0.0012), glutamate/AA (*r* = 0.27, *p =* 0.00039), asparagine/AA (*r* = −0.15, *p =* 0.048), glutamine/AA (*r* = −0.20, *p =* 0.010), tyrosine/AA (*r* = 0.17, *p =* 0.023), and proline/AA (*r* = −0.16, *p =* 0.042).

**Figure 6:**
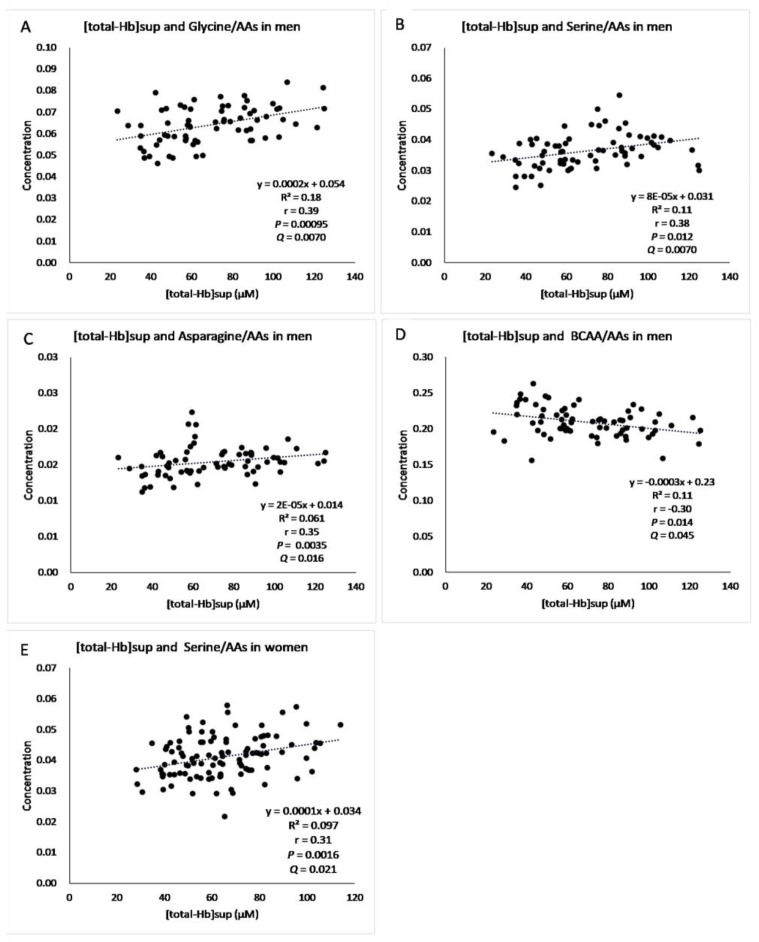
Significant relationships between [total−Hb]sup and glycine/amino acids (AAs) in men (**A**), serine/AAs in men (**B**), asparagine/AAs in men (**C**), branched-chain amino acid (BCAA)/AAs in men (**D**), serine/AAs in women (**E**) when adjusted by the false discovery rate (*Q* < 0.05). [total-Hb]sup, total hemoglobin concentration in the supraclavicular region.

**Figure 7:**
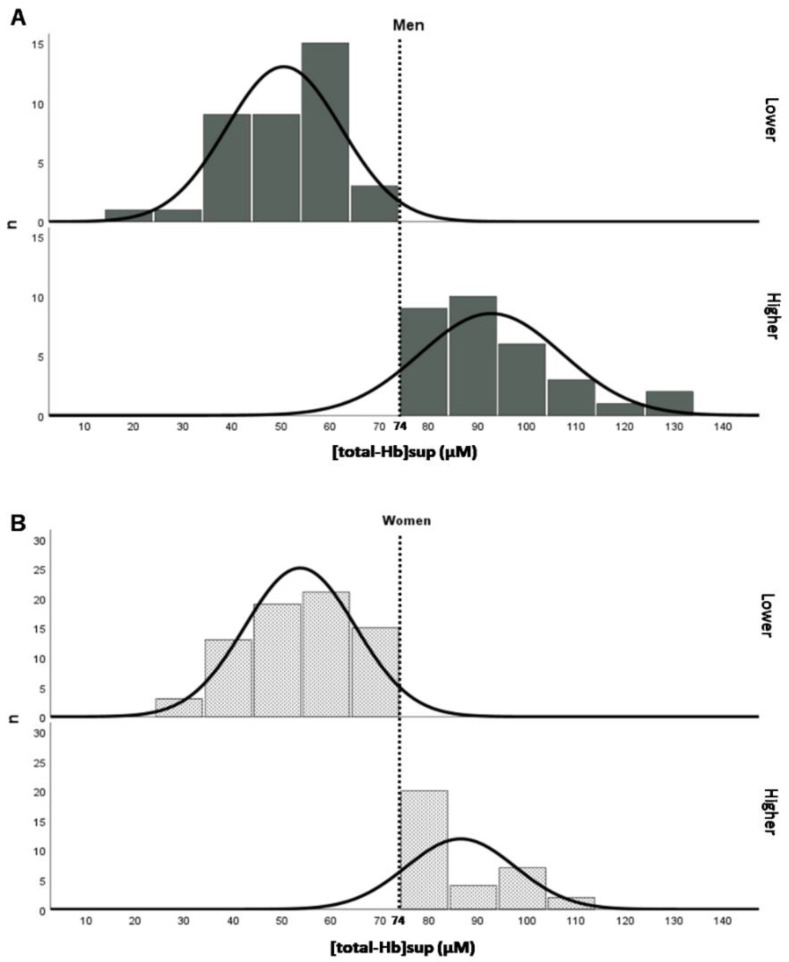
Histograms showing the [total-Hb]sup, a parameter of brown adipose tissue density (BAT-d), in men (**A**) and women (**B**) according to whether BAT was higher (>74 µM) or lower (<74 µM). [total-Hb]sup, total hemoglobin concentration in the supraclavicular region.

**Figure 8:**
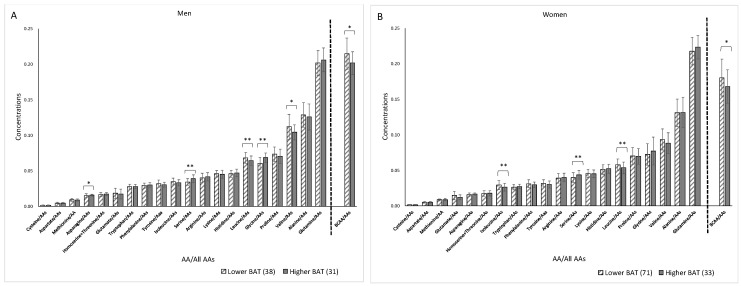
Differences in concentrations in amino acids (AAs) between higher BAT-d (>74 µM of [total-Hb]sup) and lower BAT-d (<74 µM of [total-Hb]sup) separately for men (**A**) and women (**B**). The numbers of participants in the higher and lower BAT-d groups were 31 and 38 in men and 33 and 71 in women, respectively. BCAAs, branched-chain amino acids; [total-Hb]sup, total hemoglobin concentration in the supraclavicular region. * *p* < 0.05, ** *p* < 0.01 in higher BAT-d (>74 µM of [total-Hb]sup) versus lower BAT-d (<74 µM of [total-Hb]sup).

Significant (*Q* < 0.05) relationships were found between [total-Hb]sup and glycine, asparagine, and BCAA concentrations in men and between [total-Hb]sup and serine concentrations in both men and women (Figure 6). Figure 7 shows a histogram of [total-Hb]sup according to whether BAT-d was higher or lower in men and women. Figure 8 shows the differences in the concentrations of AAs between higher and lower BAT-d separately for men and women. Significant differences were observed in the concentrations of asparagine, serine, leucine, glycine, valine, and BCAA in men and in the concentrations of isoleucine, serine, leucine, and BCAA in women according to whether the BAT-d was higher or lower. MLR analysis with plasma AA concentrations as the independent variable correlated significantly with higher or lower BAT-d in men (*r* = 0.73, *p =* 0.015) but not in women (*r* = 0.58, *p =* 0.079) (Table S1). MLR that included both plasma AA concentrations and anthropometric parameters, including age, %BF, and VFA as independent variables revealed significant correlations with higher or lower BAT-d in men (*r* = 0.82, *p =* 0.0070) and in women (*r* = 0.70, *p =* 0.020) (Table S2); when using both plasma AA and anthropometric parameters, the correlation coefficient was higher than that when using only anthropometric parameters for both men (*r* = 0.54, *p =* 0.0020) and women (*r* = 0.43, *p =* 0.0051) (Table S3). 

**Figure 9:**
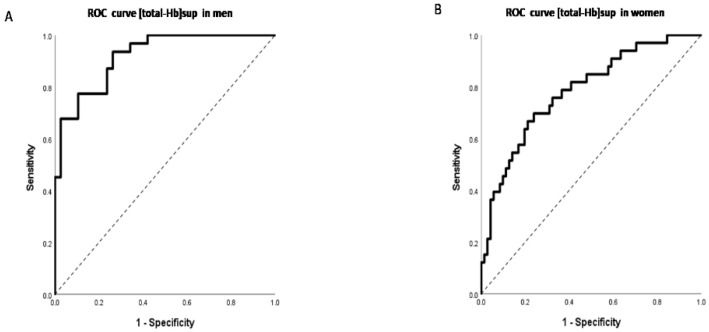
Receiver-operating characteristic (ROC) curves for total hemoglobin in the supraclavicular region ([total-Hb]sup) in men (**A**) and women (**B**). The area under the ROC curve determined by [total-Hb]sup closest to (0, 1) for the predicted formula determined by the amino acid (AA) and anthropometric parameters was selected.

The area under the ROC curve determined by [total-Hb]sup to be closest to (0, 1) for the predicted formula determined by the AAs and anthropometric parameters provided an area under the ROC curve (AUC) of 0.92 (95% confidence interval [CI] 0.86–0.98, *p* < 0.001) in men and 0.78 (95% CI 0.69–0.88, *p* < 0.001) in women if the cut-off value of total Hb was set to 74.0 μM. The sensitivity was 94% with a specificity of 74% in men and 70% with a specificity of 76% in women (Figure 9A,B).

## 4. Discussion

In this study, when the data were corrected by the false discovery rate to yield a *Q*-value, we found significant positive correlations of glycine, serine, and asparagine concentrations with [total-Hb]sup in men, a significant negative correlation of the BCAA concentration with [total-Hb]sup in men, and a significant positive correlation of the serine concentration with [total-Hb]sup in women. The precision for prediction of higher or lower BAT-d was greater in both sexes when the independent variable included both anthropometric parameters and plasma AA concentrations (men, *r* = 0.82; women, *r* = 0.70) than when plasma AA concentrations were used alone (men, *r* = 0.73; women, *r* = 0.58). This finding indicates that a combination of anthropometric parameters and plasma AA concentrations could serve as a reliable biomarker for discriminating higher from lower BAT-d. 

AAs are essential for protein synthesis in the body, and AA supplementation has been reported to have beneficial effects on muscle growth and function and on energy metabolism [26,27]. By contrast, elevated plasma BCAA levels have been found to have an adverse effect on glucose metabolism and to be associated with type 2 diabetes in humans and rodents [28,29]. The amount of BCAAs increases in the presence of insulin-resistant polymorphisms [30]. Mitochondrial BCAA enzymes in white adipose tissue are decreased under conditions of obesity and diabetes, suggesting that adipose tissue regulates BCAA levels in the blood [31,32]. Furthermore, a recent study demonstrated a significant negative correlation of BAT activity with a cold-induced reduction in the concentration of serum BCAAs, including valine and leucine, when examined in men and in those of valine, leucine, and isoleucine in obese mice [17]. In this study, we were attempting to identify a clinically practical and simple biomarker for BAT-d and therefore did not include 2 h of cold exposure. However, our results are similar to those obtained in a previous study that reported significant (*p* < 0.05) negative correlations of [total-Hb]sup with BCAA concentrations in both men and women that remained significant for men (*Q* = 0.045) but not for women (*Q* = 0.133) after correcting for the false discovery rate. 

We found that only plasma serine remained significantly correlated with [total-Hb]sup (*Q* < 0.05) in both men and women. Insulin resistance has been associated with a decrease in arginine, aspartate, and serine concentrations and an increase in proline and tyrosine concentrations, independent of age. Expression of phosphoserine aminotransferase 1, which is involved in the synthesis of serine, is reduced in mice with diabetes induced by a high-fat diet [33]. Serine is a nonessential AA, which can be synthesized in the human body from other metabolites, such as glycine, the plasma concentration of which was only significantly (*Q* < 0.05) correlated with [total-Hb]sup in men in this study. By contrast, under insulin-resistant conditions, metabolic utilization of serine for gluconeogenesis and production of glutathione from glycine may be higher, resulting in a decrease in the plasma serine concentration [34]. It is interesting that BCAA was inversely correlated with BAT-d, whereas glycine was positively correlated with BAT-d. A previous report indicates that the concentration of glycine is decreased in obese individuals and that BCAAs are a source of glycine synthesis [35]. Therefore, the results of the present study suggest that BAT contributes to catabolism of BCAAs as a metabolic sink, and the catabolized BCAAs may be used for synthesis of glycine, thereby involving insulin resistance [36]. Overall, it is reasonable to conclude that attenuated plasma serine and glycine concentrations are associated with lower BAT-d in both men and women.

We examined the relationship between plasma AA profiles under thermoneutral conditions and NIR_TRS_-determined BAT-d in our large sample of men and women. MLR, with plasma AAs as the independent variable, revealed a significant correlation of AA concentrations with higher or lower BAT-d in men (*r* = 0.73, *p* = 0.015) but not in women (*r* = 0.58, *p* = 0.079). A previous study found that anthropometric parameters were independently correlated with [total-Hb]sup [19], with a correlation coefficient of *r* = 0.54 (*p* = 0.0020) in men and *r* = 0.43 (*p* = 0.0051) in women, which is in line with our present data. Therefore, we examined whether the precision for predicting BAT-d could be increased by using a combination of anthropometric parameters and plasma AA concentrations as the independent variable and found better correlations with higher or lower BAT-d for both men (*r* = 0.82, *p* = 0.0070) and women (*r* = 0.70, *p* = 0.020).

Despite the relevance of biomarkers for evaluation of BAT, each methodology has several limitations [3]. Our results indicate that a combination of anthropometric parameters and plasma AA concentrations provides a clinically practical and simple biomarker for BAT-d with low cost and no ionizing radiation exposure that is not time-consuming to perform and does not require acute cold exposure to stimulate BAT. Previous studies have identified other plasma biomarkers, including miRNA122 [37], LysoPC-acyl C16:0 [38], 12,13-diHOME [39], and androgens in men [19], to be potential candidates for identifying the characteristics of BAT when combined with anthropometric parameters as in the present study.

In another study, cold-induced FDG-PET/CT measurements showed a significant correlation between [total-Hb]sup under thermoneutral conditions and parameters of BAT activity/volume [13]. In our study, we also confirmed a significant correlation between cold-induced thermogenesis, which is an indicator of BAT activity [18], and [total-Hb]sup. Furthermore, a longitudinal study found that [total-Hb]sup and ^18^FDG-PET/CT parameters increased in parallel during chronic intake of thermogenic capsinoids, which are known to increase the mass and activity of BAT, and decreased after cessation of intake [40,41]. Therefore, we believe that [total-Hb]sup can be used to evaluate BAT-d under thermoneutral conditions.

The main limitation of this study is that when collecting blood samples, we did not control for the menstrual cycle. Although menstrual cycle was expected to show larger variations in plasma AA concentrations in women, we did not find a significant difference in variations (SD) in plasma AA concentrations between men and women. Although there has been a report of an age-related decrease in [total-Hb]sup that may influence plasma AA concentrations, we did not take this into account in this study. Furthermore, we found a significant biomarker only in lean healthy individuals in this study. Future research should include obese individuals and patients with cardiometabolic disease. Finally, given that the correlation coefficient for [total-Hb]sup and cold-induced FDG-PET/CT measurements was moderate (*r* = 0.74) [13], a detailed validation study should be conducted using FDG-PET/CT methodology in the future. The requirement for venous blood collection and the use of mass spectrometry represent additional challenges for using plasma AA profiles to predict BAT-d in clinical settings.

## 5. Conclusions

When the data obtained in this study were corrected by the false discovery rate, we found a significant positive correlation of glycine, serine, and asparagine concentrations and a negative correlation of the BCAA concentration with [total-Hb]sup in men. Moreover, there was a significant positive correlation of the serine concentration with [total-Hb]sup in women and in all study participants. A combination of anthropometric parameters and plasma AA concentrations may serve as a reliable biomarker for discriminating higher from lower BAT-d in both men (*r* = 0.82) and women (*r* = 0.70). The results of our study indicate that BAT-d can be estimated using the biomarker determined in this study at low cost and with no ionizing radiation exposure. Additional benefits of this biomarker are that it is not time-consuming to perform and does not require acute cold exposure to stimulate BAT.

## Figures and Tables

**Table 1:** Demographic and anthropometric characteristics of the study participants.

	Men (*n* = 69)	Women (*n* = 104)	All (*n* = 173)	*p*-Value *
Age (years)	38.12 ± 10.11	37.86 ± 7.30	37.96 ± 8.51	0.999
Height (cm) **	172.53 ± 5.69	159.18 ± 5.46	164.50 ± 8.58	<0.001
Body weight (kg) **	69.54 ± 10.42	54.96 ± 8.62	60.78 ± 11.77	<0.001
BMI **	23.38 ± 3.48	21.67 ± 3.00	22.35 ± 3.30	0.001
%BF (%) **	20.92 ± 6.44	28.74 ± 6.04	25.60 ± 7.28	<0.001
VFA (cm^2^) **	66.51 ± 39.00	34.37 ± 23.62	47.27 ± 34.45	<0.001
[total-Hb]sup (µM)	69.48 ± 24.76	64.06 ± 18.95	66.22 ± 21.55	0.216

Values are shown as the mean ± standard deviation. * *p* < 0.05, ** *p* < 0.01 in men versus women. %BF, percentage of body fat; BMI, body mass index; [total-Hb]sup, total hemoglobin concentration in the supraclavicular region (an indicator of brown adipose tissue density); VFA, visceral fat area.

**Table 2:** Correlation between amino acid concentrations and total hemoglobin concentration in the supraclavicular region.

	Men				Women			
				Valine, Leucine, and Isoleucine (BCAAs)				Valine, Leucine, and Isoleucine (BCAAs)
	*r*	*p*-Value	*Q*-Value	*Q*-Value	*r*	*p*-Value	*Q*-Value	*Q*-Value
Glycine/AAa	0.39 **	0.00095	0.00700	0.00500	0.14	0.160	0.33	0.36
Alanine/AAs	−0.086	0.48	0.63	0.55	−0.0043	0.966	0.97	0.97
Valine/AAs	−0.29 *	0.016	0.067		−0.18	0.065	0.20	
Leucine/AAs	−0.025 *	0.037	0.13		−0.23 *	0.018	0.080	
Isoleucine/AAs	−0.19	0.12	0.32		−0.23 *	0.019	0.080	
Cysteine/AAs	−0.11	0.35	0.52	0.47	−0.06	0.564	0.74	0.77
Methionine/AAs	0.047	0.70	0.78	0.67	0.14	0.172	0.33	0.36
Serine/AAs	0.38 **	0.0012	0.0070	0.0050	0.31 **	0.0016	0.021	0.019
Homoserine + threonine/AAs	0.23	0.057	0.17	0.15	0.071	0.476	0.67	0.70
Aspartate/AAs	0.038	0.76	0.80	0.68	−0.090	0.365	0.59	0.63
Glutamate/AAs	−0.15	0.22	0.44	0.41	−0.19 *	0.049	0.17	0.19
Asparagine/AAs	0.35 **	0.0035	0.016	0.012	0.14	0.143	0.33	0.36
Glutamine/AAs	0.170	0.16	0.38	0.37	0.048	0.630	0.77	0.78
Arginine/AAs	0.075	0.54	0.63	0.55	0.12	0.241	0.42	0.46
Lysine/AAs	−0.14	0.24	0.44	0.41	0.016	0.869	0.91	0.92
Histidine/AAs	0.094	0.44	0.62	0.55	0.044	0.657	0.77	0.78
Phenylalanine/AAs	0.140	0.25	0.44	0.41	0.025	0.801	0.89	0.90
Tyrosine/AAs	−0.130	0.29	0.46	0.42	−0.079	0.427	0.64	0.68
Tryptophan/AAs	−0.080	0.52	0.63	0.55	0.24 *	0.015	0.08	0.10
Proline/AAs	−0.031	0.80	0.80	0.68	−0.14	0.167	0.33	0.36
BCAAs/AAs	−0.30 *	0.014		0.045	−0.22 *	0.028		0.13

*p*-values were adjusted using the false discovery rate method (Benjamini and Hochberg) to yield *Q*-values. The *Q*-value was calculated from the AA and total hemoglobin concentrations. The BCAAs were evaluated together and for leucine, isoleucine, and valine individually (according to Figure 1, Figure 2, Figure 3, Figure 4 and Figure 5). BCAAs, branched-chain amino acids. * *p* < 0.05, ** *p* < 0.01 in *p*-Values.

## Data Availability

The data presented in this study are available on request from the corresponding author. The data are not publicly available for privacy reasons.

## Supplementary Materials

The following are available online at https://www.mdpi.com/article/10.3390/jcm10112339/s1, Table S1. Results of multiple logistic regression analysis with plasma AA concentrations as the independent variable showed significant correlations with higher or lower BAT-d in men and women, Table S2. Results of multiple logistic regression analysis with plasma AA concentrations and anthropometric parameters, including age, %BF, and VFA, as independent variables showed significant correlations with higher or lower BAT-d in men and women, Table S3. Results of multiple logistic regression analysis with anthropometric parameters alone, including age, %BF, and VFA, as independent variables revealed significant correlations with higher or lower BAT-d in men and women.
