# Supplementary material for: Correlation of Plasma Amino Acid and Anthropometric Profiles with Brown Adipose Tissue Density in Humans
Source: J Clin Med. 2021 May 27;10(11):2339. doi: 10.3390/jcm10112339 (PMC8199240; doi:10.3390/jcm10112339)
Supplement: Supplementary file 1 [file jcm-10-02339-s001.zip › Table S1.pdf]

**Table S1.** Results of multiple logistic regression analysis with plasma AA concentrations as the independent variable showed significant correlations with higher or lower BAT-d in men and women

| | Men | | | Women | | |
|---|---|---|---|---|---|---|
| | *P*-value | B | ExP(B) | *P*-value | B | ExP(B) |
| **Glycine/AAs** | **0.043** | 137.82 | $7.17×10^{59}$ | 0.657 | -13.10 | $2.05×10^{-6}$ |
| **Alanine/AAs** | 0.90 | -5.20 | 0.0055 | 0.469 | -20.12 | $1.83×10^{-9}$ |
| **Valine/AAs** | 0.70 | -21.15 | $6.54×10^{-10}$ | 0.834 | -6.61 | $1.34×10^{-3}$ |
| **Leucine/AAs** | 0.70 | -34.46 | $1.08×10^{-15}$ | 0.481 | -55.06 | $1.22×10^{-24}$ |
| **Isoleucine/AAs** | 0.53 | 80.32 | $7.61×10^{34}$ | 0.311 | 104.55 | $3.93×10^{-46}$ |
| **Cysteine/AAs** | 0.31 | -1372.49 | - | 0.594 | -496.21 | $3.15×10^{-216}$ |
| **Methionine/AAs** | 0.57 | -213.02 | $3.08×10^{-93}$ | 0.290 | 281.54 | $1.87×10^{122}$ |
| **Serine/AAs** | 0.037 | 169.38 | $3.63×10^{73}$ | 0.251 | 63.56 | $4.00×10^{27}$ |
| **Homoserine + Threonine/AAs** | 0.14 | 331.32 | $7.80×10^{143}$ | 0.236 | -127.09 | $6.38×10^{-56}$ |
| **Aspartate/AAs** | 0.31 | 539.46 | $1.93×10^{234}$ | 0.681 | 117.32 | $8.98×10^{50}$ |
| **Glutamate/AAs** | 0.30 | 91.11 | $3.72×10^{39}$ | 0.158 | -105.66 | $1.30×10^{-46}$ |
| **Asparagine/AAs** | 0.36 | -223.39 | $9.58×10^{-98}$ | 0.186 | 210.41 | $2.40×10^{91}$ |
| **Glutamine/AAs** | 0.97 | 1.37 | 3.94 | 0.572 | -16.26 | $8.67×10^{-8}$ |
| **Arginine/AAs** | 0.45 | 56.02 | $2.13×10^{24}$ | 0.792 | -17.68 | $2.10×10^{-8}$ |
| **Lysine/AAs** | 0.76 | -28.35 | $4.89×10^{-13}$ | 0.103 | -111.03 | $6.01×10^{-49}$ |
| **Histidine/AAs** | 0.37 | 88.03 | $1.70×10^{38}$ | 0.872 | -8.87 | $1.40×10^{-4}$ |
| **Phenylalanine/AAs** | **0.036** | 307.70 | $4.29×10^{133}$ | 0.093 | -141.72 | $2.82×10^{-62}$ |
| **Tyrosine/AAs** | 0.53 | -78.95 | $5.13×10^{-35}$ | 0.725 | -25.97 | $5.25×10^{-12}$ |
| **Tryptophan/AAs** | 0.36 | 122.45 | $1.51×10^{53}$ | 0.108 | 158.29 | $5.54×10^{68}$ |
