# Supplementary material for: Correlation of Plasma Amino Acid and Anthropometric Profiles with Brown Adipose Tissue Density in Humans
Source: J Clin Med. 2021 May 27;10(11):2339. doi: 10.3390/jcm10112339 (PMC8199240; doi:10.3390/jcm10112339)
Supplement: Supplementary file 1 [file jcm-10-02339-s001.zip › Table S2.pdf]

Table S2. Results of multiple logistic regression analysis with plasma AA concentrations and anthropometric parameters, including age, %BF, and VFA, as independent variables showed significant correlations with higher or lower BAT-d in men and women

	Men			Women		
	P-value	B	ExP(B)	*P*-value	B	ExP(B)
**Glycine/AAs**	0.18	133.20	7.02×10^{57}	0.37	-41.81	6.95×10^{-19}
**Alanine/AAs**	0.68	27.89	1.30×10^{12}	0.54	-25.85	5.93×10^{-12}
**Valine/AAs**	0.44	-59.01	2.36×10^{-26}	0.57	25.93	1.83×10^{11}
**Leucine/AAs**	0.45	94.20	8.11×10^{40}	0.10	-176.29	2.76×10^{-77}
**Isoleucine/AAs**	0.20	363.64	8.45×10^{157}	0.46	-95.37	3.82×10^{-42}
**Cysteine/AAs**	0.77	548.23	1.24×10^{238}	0.12	-2231.44	-
**Methionine/AAs**	0.46	-487.23	2.51×10^{-212}	0.084	573.72	1.46×10^{249}
**Serine/AAs**	0.16	169.99	6.66×10^{73}	0.36	71.49	1.12×10^{31}
**Homoserine + Threonine/AAs**	**0.038**	680.43	3.20×10^{295}	0.40	-110.74	8.079×10^{-49}
**Aspartate/AAs**	0.24	1297.44	-	0.59	179.98	1.45×10^{78}
**Glutamate/AAs**	0.40	117.79	1.43×10^{51}	0.21	-134.36	4.43×10^{-59}
**Asparagine/AAs**	0.76	-125.30	3.84×10^{-55}	0.46	145.82	2.12×10^{63}
**Glutamine/AAs**	0.76	22.87	8.55×10^{9}	0.91	-4.88	7.57×10^{-3}
**Arginine/AAs**	0.36	-133.02	1.69×10^{-58}	0.19	-131.86	5.42×10^{-58}
**Lysine/AAs**	0.32	162.79	5.01×10^{70}	0.72	-35.28	4.79×10^{-16}
**Histidine/AAs**	0.13	293.47	2.84×10^{127}	0.65	-33.88	1.94×10^{-15}
**Phenylalanine/AAs**	0.13	357.61	2.03×10^{155}	0.25	-112.83	9.99×10^{-50}
**Tyrosine/AAs**	0.22	-208.14	4.05×10^{-91}	0.45	76.89	2.48×10^{33}
**Tryptophan/AAs**	0.20	269.59	1.21×10^{177}	0.40	112.017	4.45×10^{48}
**Age**	0.26	0.08	1.086	0.97	0.0021	1.0021
**%BF**	**0.052**	-0.38	0.69	0.036	-0.19	0.83
**VFA**	0.62	-0.01	0.99	0.76	0.0063	1.0063
