# Supplementary material for: Correlation of Plasma Amino Acid and Anthropometric Profiles with Brown Adipose Tissue Density in Humans
Source: J Clin Med. 2021 May 27;10(11):2339. doi: 10.3390/jcm10112339 (PMC8199240; doi:10.3390/jcm10112339)
Supplement: Supplementary file 1 [file jcm-10-02339-s001.zip › Table S3.pdf]

Table S3. Results of multiple logistic regression analysis with anthropometric parameters alone, including age, %BF, and VFA, as independent variables revealed significant correlations with higher or lower BAT-d in men and women

	**Men**			**Women**		
	***P*-value**	**B**	**ExP(B)**	***P*-value**	**B**	**ExP(B)**
**Age**	0.58	0.018	1.018	0.35	-0.037	0.96
**%BF**	0.18	-0.10	0.90	0.017	-0.15	0.86
**VFA**	0.10	-0.021	0.98	0.99	-0.00012	0.999
